# Comparison of hyperbilirubinemia incidence between the newborns of zinc-taking and non-zinc-taking mothers during the third trimester of pregnancy

**DOI:** 10.22088/cjim.12.4.52

**Published:** 2021

**Authors:** Hassan Boskabadi, Gholamali Maamouri, Maryam Zakerihamidi, Atiyeh Mohammadzadeh Vatanchi, Mohammad Sokhtanloo, Marzieh Sadat Mousavi, Sara Ghahremani, Fatemeh Bagheri

**Affiliations:** 1 Department of Pediatrics, Faculty of Medicine, Mashhad University of Medical Sciences, Mashhad, Iran; 2 Department of Midwifery, Faculty of Medical Sciences, Tonekabon Branch, Islamic Azad University, Tonekabon, Iran; 3 Department of Gynecology, Faculty of Medicine, Mashhad University of Medical Sciences, Mashhad, Iran; 4 Department of Biochemistry, Faculty of Medicine, Mashhad University of Medical Sciences, Mashhad, Iran; 5 Department of Nursing, Faculty of Medical Sciences, Mashhad Branch, Islamic Azad University, Mashhad, Iran

**Keywords:** Infants, Hyperbilirubinemia, Zinc, Umbilical cord, Jaundice, Mothers

## Abstract

**Background:**

Identification and control of the risk factors for hyperbilirubinemia can reduce the incidence and complications of this condition. Serum zinc level in newborns is reported to be one of the factors affecting the severity and incidence of neonatal hyperbilirubinemia. Therefore, the present study was conducted to compare hyperbilirubinemia incidence in neonates of zinc-taking and non-zinc-taking mothers.

**Methods:**

In this observational study, we compared the incidence of hyperbilirubinemia during the first week of birth between newborns whose mothers had received zinc supplement during the third trimester of pregnancy (case group) with those whose mothers had not taken zinc supplement (control group). The checklist of newborns’ conditions in both case and control groups was completed based on the obtained data regarding the infants, mothers, and laboratory findings. Data was analyzed using chi-squared test and *t*-test.

**Results:**

The mean serum levels of zinc were 79.76±15 mg/dl and 70.93±15.67mg/dl in mothers who had received zinc during the third trimester and those who had not taken zinc supplement, respectively. The mean serum level of zinc in newborns who underwent phototherapy was 41.68±9.21 mg/dl, while it was 68.53±20.85 mg/dl in neonates who did not receive phototherapy. In addition, 36% of the neonates whose mothers had not received zinc required phototherapy, while only 11% of newborns whose mothers had taken zinc supplement received phototherapy.

**Conclusion:**

Zinc consumption during the third trimester of pregnancy increased the serum zinc level in both newborns and mothers and reduced the incidence and severity of idiopathic hyperbilirubinemia requiring treatment.

Jaundice is by far the most common cause of neonatal hospitalization during the first month of life. Phototherapy is the mainstay of treatment for this disease, and failure in the timely treatment of this condition can cause serious complications such as kernicterus, which leads to lifelong disabilities (1). Identification of the risk factors for hyperbilirubinemia and controlling them can lower the incidence and complications of this disease. In general, preventing the onset of severe neonatal hyperbilirubinemia is more reliable than the conventional treatments such as phototherapy and/or blood exchange transfusion. One of the influential factors affecting the incidence and severity of neonatal hyperbilirubinemia is the serum level of zinc in newborns (2). Mild zinc deficiency, on the other hand, is associated with intrauterine growth restriction, low birth weight, and pre-term and post-term labor (3, 4).

The level of zinc in the umbilical cord blood was found to be lower in icteric infants (newborns with hyperbilirubinemia) as compared to healthy neonates, suggesting that zinc deficiency in newborns can be a risk factor for neonatal hyperbilirubinemia (2). However, the results of a study conducted by Mohammadzadeh et al. indicated that the daily use of 20 mg zinc sulfate had no protective effects against hyperbilirubinemia in very-low-birth-weight (VLBW) pre-term newborns. Zinc supplementation also did not influence the duration of phototherapy (5). Moreover, in a study carried out by Maamouri et al. the prophylactic administration of zinc during the first five days of life failed to impact the incidence and severity of hyperbilirubinemia (6), which is in line with the findings of a systematic review performed by Chaffee and King (12). Review of the literature shows that most studies examining the influence of zinc supplementation on neonatal bilirubin have studied infants rather than their mothers. To the best of our knowledge, yet no study has investigated the impact of maternal zinc consumption on neonatal hyperbilirubinemia, which is one of the most common neonatal conditions in Asia. Therefore, in the present study we sought to compare the incidence of hyperbilirubinemia between newborns of zinc-taking and non-zinc-taking mothers.

## Methods

In this observational study, we compared the incidence of hyperbilirubinemia during the first week of life among 169 newborns of zinc-taking and non-zinc-taking mothers. The newborns were selected using the convenience sampling method from among infants admitted to the emergency unit or neonatal intensive care unit (NICU) of Ghaem Hospital in Mashhad, Iran, during March 2014 to March 2019. The hospitalized newborns with hyperbilirubinemia were considered as the treatment-requiring group and those who did not receive treatment or were not affected by hyperbilirubinemia were considered as the no treatment group. Ethical approval (IR.MUMS.MEDICAL.REC.1392.33) was obtained from the Ethics Committee of the Mashhad University of Medical Sciences (Grant number: 910684). After obtaining a written informed consent, 190 pregnant women were assigned to the two groups of case (taking 25 µg/day zinc during the third trimester of pregnancy) and control (not using zinc supplement). Thereafter, maternal and cord blood serum zinc levels were evaluated. For this purpose, 0.5 cc of blood was drawn, serum was then extracted and sent to the laboratory in either dark or covered tubes for zinc-content measurement. Serum tubes and ends of samplers were rinsed using acid and deionized water prior to sampling to ensure they are devoid of any trace elements. The samples were centrifuged for 10 min at 1000 rpm, and then serum was collected in polyethylene tubes which were well-rinsed with acid. The tubes were stored at -70°C until analysis. Zinc analysis was carried out by atomic absorption spectrophotometry using air/acetylene fuel. 

Newborns were followed-up during their first week of life for jaundice. Exclusion criteria entailed: 1) irregular zinc consumption (not using the supplement more than two days a week), 2) twins and multiple pregnancy, 3) diabetic mothers, 4) placenta previa, 5) polyhydramnios, 6) maternal Rh-negative blood groups, 7) evidence of hemolysis in newborns with hyperbilirubinemia, 8) ABO incompatibility, 9) glucose-6-phosphate dehydrogenase (G6PD) deficiency, and 8) failure to follow-up. The checklist of newborns’ conditions in both case and control groups was completed based on the obtained data regarding infants, mothers, and laboratory analyses. Neonatal information included age, gender, birth weight, weight on admission, and hyperbilirubinemia, maternal characteristics consisted of zinc supplement consumption and route of delivery, and laboratory findings comprised bilirubin level, maternal and neonatal zinc levels, hematocrit, and platelet count. Laboratory tests or any other Para clinical studies were ordered by a neonatologist, and the results were recorded without any interventions. Transcutaneous bilirubinometry was carried out using a jaundice-meter at least three times prior to neonates’ discharge (on the third to fifth, and seventh days of life), and serum bilirubin was evaluated if needed. The phototherapy group was compared to the no-treatment group in terms of mean bilirubin level, and the zinc-taking and non-zinc-taking mothers were compared with regard to the mean serum zinc levels. 

**Statistical Analysis: **Initially, the obtained results were described using tables and figures, the newborns in the case and control groups were then compared using chi-squared test and *t*-test in SPSS Version 20. A *p-*value of less than 0.05 was considered statistically significant.

## Results

Of the188 mothers, six were excluded from the study due to irregular zinc consumption and three owing to taking other zinc-containing drugs. In addition, four newborns were hospitalized for reasons other than hyperbilirubinemia, and six mothers did not complete their newborns’ follow-up. In the control group, 34 (41.5%) newborns were males and 48 (58.5%) were females, whereas in the case group, 53 (61%) neonates were boys and 33 (39%) were girls.

The mean serum zinc levels of the mothers in the case and control groups were 79.76±15.69 mg/dl and 70.93±15.67 mg/dl, respectively (P=0.076), and serum bilirubin levels of their newborns were obtained as 11.12±3.64 mg/dl and 15.15±2.03 mg/dl, respectively (p=0.019). Of the 169 newborns, 129 (76.3%) did not need treatment and 40 (23.7%) underwent phototherapy. Hyperbilirubinemia was detected in 100 (59.2%) newborns which necessitated treatment in 40 (23.7%) of them, 38 of whom were hospitalized and underwent phototherapy and the other two received phototherapy at home. Hospitalization and phototherapy were not indicated for the remaining 60 newborns diagnosed with hyperbilirubinemia. The mean serum levels of bilirubin in newborns in the treatment-requiring and no treatment groups were 18.21±3.31 mg/dl and 10.32±2.12 mg/dl, respectively (P=0.000). In addition, the mean serum zinc level of infants who received phototherapy was lower that of the no treatment group (P=0.000; table 1).

In this study, newborns of zinc-taking and non-zinc-taking mothers were significantly different in terms of the received treatment (P=0.009). In other words, the rate of need for phototherapy was higher in newborns of non-zinc-taking mothers than in infants of the case group (table 2).

**Table1:** Comparison of newborns of the case and control group in terms of neonatal variables

**Groups**	**No treatment** **infants 129 (76.3%)**	**Treatment-requiring** **infants 40 (23.7%)**	Significance level*
Variables			
Bilirubin (mg/dl)	10.32±2.12	18.21±3.31	0.000
Zinc (maternal)	76.23±15.93	72.83±14.61	0.459
Zinc (neonatal)	68.53±20.85	41.68±9.21	0.000

*values are in mean±standard deviation

**Table 2:** Comparison of newborns of the case and control groups in terms of neonatal variables

**Groups**	**Control group** **infants 83 (49.4%)**	**Intervention group (zinc-taking mothers)** **infants 86 (50.6%)**	***P*** **-value** **(Chi-square)**
Variables			
No treatment	53(63.9%)	76(88.4%)	0.009
Phototherapy	30(36.1%)	10(11.6%)	

## Discussion

In the present study, zinc consumption during the third trimester of pregnancy increased the serum level of zinc; however, this increase was not significant. A systematic review revealed that maternal consumption of zinc increases maternal serum level of zinc in the late pregnancy period (7). The short duration and low doses of zinc consumption by mothers are the probable reasons responsible for the insignificant increase in serum zinc level among the zinc-taking mothers. Another reason might be the high rate of zinc transport to supply the zinc storage of the fetal liver.

In the present study, maternal zinc consumption increased the fetal serum level of zinc up to 1.6 times. Based on the results of a study, maternal and fetal serum zinc levels are positively correlated (8). The binding affinity of mother's blood to zinc decreases during pregnancy facilitating the active transport of zinc from the mother to the fetus, which elevates serum zinc level in the umbilical cord blood (9, 10). Therefore, the increased zinc level in newborns of zinc-taking mothers might be due to the effective placental transport of zinc. According to the results of the current study, maternal zinc consumption decreased both the severity of hyperbilirubinemia and the incidence of treatment-demanding idiopathic hyperbilirubinemia in newborns. The association of zinc level with neonatal hyperbilirubinemia is pertinent to the ability of zinc in reaching the intestine, where bilirubin is absorbed into the bloodstream leading to decreased enterohepatic cycle of bilirubin (11). Zinc sulfate reduces bilirubin absorption and its enterohepatic cycle by bonding to unconjugated bilirubin (12). Based on the results of the present study, bilirubin levels were lower in newborns whose mothers consumed zinc supplements during pregnancy. The results of a study suggested that serum zinc levels in newborns with hyperbilirubinemia were lower as compared to healthy newborns (2). A study conducted in Turkey demonstrated significantly lower serum zinc levels in newborns with hyperbilirubinemia and their mothers as compared to control group newborns and mothers (13). 

It is estimated that about 50% of the women of the reproductive age suffer from zinc deficiency. Low maternal serum zinc level is associated with reduced zinc transport to the fetus and developmental malformations (14). The findings of a study carried out by Vitek et al. revealed that obtaining oral zinc salts reduced bilirubin levels in icteric mice through enterohepatic cycle suppression; therefore, it can be recommended for the management of severe unconjugated hyperbilirubinemia (15). Zinc salts and other elements, such as strontium, prevent hyperbilirubinemia by inhibiting heme oxygenase (16). Zinc is the inhibitor for heme oxygenase enzyme both *in-vitro* and *in-vivo*. Bilirubin and carbon-monoxide levels decline 1-6 hours after the administration of 4 µg/kg of zinc (17). According to a study performed by Mafinejad et al., oral administration of 10 mg/day zinc sulfate to pre-term neonates weighing less than 1800 g reduced serum bilirubin level and the need for phototherapy (18). 

The results of a study conducted by Hashemian et al. signified that 10 mg/day oral consumption of zinc sulfate led to the reduction of serum bilirubin level and the duration of phototherapy. Therefore, it is proposed to use zinc sulfate along with phototherapy as a safe medication and a nutritional trace element effective in non-hemolytic neonatal hyperbilirubinemia (19). In a study performed by Kumar et al., daily consumption of 10 mg of zinc sulfate failed to affect neonatal idiopathic hyperbilirubinemia (20). The role of zinc in the prevention of neonatal hyperbilirubinemia is not supported according to the results of a systematic review. There only exists one study indicating the role of zinc in the reduction of mean serum bilirubin level and the need for phototherapy, and the rest of studies did not report any positive effects for zinc. These studies did not confirm the effects of zinc on the duration and incidence of hyperbilirubinemia, the age of phototherapy onset, or any other unfavorable serious conditions (21). Therefore, former studies suggest that despite zinc deficiency in icteric newborns, zinc consumption has failed to reduce the severity and incidence of hyperbilirubinemia. The insufficient duration by infant might be one of the probable reasons for not responding to zinc consumption. 

In the current study, bilirubin level in newborns of mothers who consumed zinc during the last trimester of their pregnancy was half of those in the control group. In other studies, serum zinc levels were significantly lower in newborns suffering from hyperbilirubinemia as compared to healthy newborns (2, 22). During the third trimester of pregnancy, fetal zinc level increases and mostly accumulates in the liver. Zinc supplement consumption by the mother during pregnancy might be helpful and leads to fetal growth and immune system maturation (23). In a study conducted by Tabrizi et al. (2014), umbilical cord levels of zinc and iron were greater during the third trimester in comparison with the maternal serum levels of zinc and iron. Moreover, maternal serum zinc level declines during pregnancy (24). This reduction can be attributed to the uneven increase in plasma volume in the mother, maternal-fetal transport of zinc, and the increased amount of iron and copper in the nutrition which compete with zinc on absorption sites (25). Zinc is a heme oxygenase inhibitor and prevents hyperbilirubinemia; therefore, the administration of 4 µg/kg of zinc to adult rats led to a decrease in carbon-monoxide and a significant decline in serum bilirubin level within 1-6 hours following treatment (17). In this study, it was not possible to perform laboratory testing for all physiological jaundice cases, which was the main limitation of the present study. 

The leading cause of neonatal hospitalization during the first month of life is neonatal hyperbilirubinemia. Maternal zinc supplementation increases maternal and fetal serum zinc levels and reduces the incidence and severity of treatment-demanding idiopathic hyperbilirubinemia. Thus, daily consumption of 25 µg of zinc is recommended to pregnant women with fetuses at high risk for neonatal hyperbilirubinemia. However, further studies are required since maternal serum levels of zinc did not show a significant difference with and without taking zinc salts probably due to the short duration of zinc consumption.
